# Discovery and Anticancer Activity of the Plagiochilins from the Liverwort Genus *Plagiochila*

**DOI:** 10.3390/life13030758

**Published:** 2023-03-10

**Authors:** Christian Bailly

**Affiliations:** 1 Institut de Chimie Pharmaceutique Albert Lespagnol (ICPAL), Faculté de Pharmacie, University of Lille, 3 rue du Professeur Laguesse, F-59006 Lille, France; christian.bailly@oncowitan.com; 2 CNRS, Inserm, CHU Lille, UMR9020-U1277-CANTHER-Cancer Heterogeneity Plasticity and Resistance to Therapies, University of Lille, F-59000 Lille, France; 3 OncoWitan, Consulting Scientific Office, Wasquehal, F-59290 Lille, France

**Keywords:** anticancer agents, aromadendrane, bryophytes, cytokinesis inhibitor, *Plagiochila* species, plagiochilins

## Abstract

The present analysis retraces the discovery of plagiochilins A-to-W, a series of seco-aromadendrane-type sesquiterpenes isolated from diverse leafy liverworts of the genus *Plagiochila*. Between 1978, with the first isolation of the leader product plagiochilin A from *P. yokogurensis*, and 2005, with the characterization of plagiochilin X from *P. asplenioides*, a set of 24 plagiochilins and several derivatives (plagiochilide, plagiochilal A-B) has been isolated and characterized. Analogue compounds recently described are also evoked, such as the plagiochianins and plagicosins. All these compounds have been little studied from a pharmacological viewpoint. However, plagiochilins A and C have revealed marked antiproliferative activities against cultured cancer cells. Plagiochilin A functions as an inhibitor of the termination phase of cytokinesis: the membrane abscission stage. This unique, innovative mechanism of action, coupled with its marked anticancer action, notably against prostate cancer cells, make plagiochilin A an interesting lead molecule for the development of novel anticancer agents. There are known options to increase its potency, as deduced from structure–activity relationships. The analysis shed light on this family of bryophyte species and the little-known group of bioactive terpenoid plagiochilins. Plagiochilin A and derivatives shall be further exploited for the design of novel anticancer targeting the cytokinesis pathway.

## 1. Introduction

Bryophytes are non-vascular plants which include thalloid and leafy liverworts, mosses and hornworts. These three lineages form a unique part of the vegetation. They are small-sized, structurally simple diversified plants able to adapt to most ecosystems on Earth [1,2]. Bryophytes and tracheophytes (non-vascular and vascular plants, respectively) derive from an ancestral land plant and diverged during the Cambrian, some 500 million years ago [3]. Bryophytes are collectively divided into three main groups: Bryophyta (mosses), Marchantiophyta (liverworts) and Anthocerotophyta (hornworts). They represent the second-largest group of land plants after angiosperms. Liverworts are particularly abundant, with some 7300 extant species [4]. The first representations of liverworts date from late antiquity [5].

Leafy (or scaly) liverworts are particularly abundant and diversified (order: Jungermanniales). They grow commonly on moist soil or damp rocks (such as thallose liverworts). In 2016, a worldwide checklist for liverworts and hornworts included 7486 species in 398 genera representing 92 families from the two phyla [6]. The genus *Plagiochila* (Plagiochilaceae) represents one of the largest groups of leafy liverworts, with more than 500 species distributed worldwide and a broad geographical amplitude, mostly in the humid tropics [7]. World Flora Online refers to 556 accepted names of *Plagiochila* species and more than 950 species including synonyms and unchecked species [8]. Another recent study refers to 1600 validly published *Plagiochila* names [9].

Despite their number and high adaptative capacities, the medicinal use of bryophytes remains relatively limited, probably because of their small size, lack of conspicuous organs such as colored fruits and flowers, and the difficulty of identification. There are, however, species with medicinal properties, such as *Conocephalum conicum* (L.) Dumort., *Polytrichum commune* and *Marchantia chenopoda* L. [10]. In the genus *Plagiochila*, a few species have also been used ethnomedicinally, such as *P. beddomei* Steph. used in the form of paste by tribe Melghat Region (India) for treating skin diseases [11,12] and *P. disticha* (Lehm. & Lindenb.) Lindenb used traditionally in Peru to treat rheumatism or to regulate menstruation [13].

**Figure 1:**
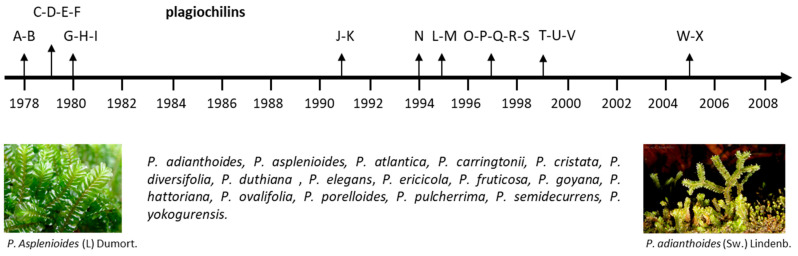
History of plagiochilins discovery. The 24 plagiochilins (A–W) have been identified and structurally characterized aver a period of 30 years. They are produced by several *Plagiochila* species, such as those indicated (a non-exhaustive list).

**Figure 2:**
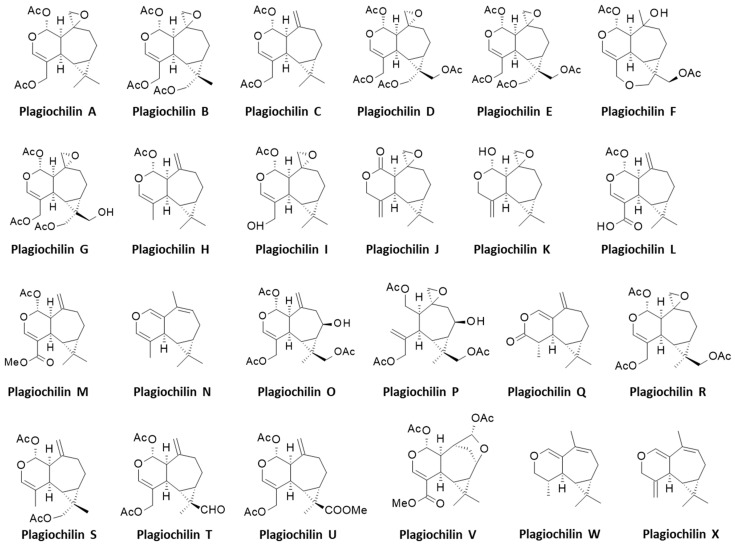
Structures of the 24 plagiochilins (A-to-W).

Diverse bioactive products have been isolated from *Plagiochila* species, including antitumor agents [14], antifungal molecules [15,16], insecticidal compounds [17] and antimicrobial products [18]. Most of the isolated bioactive compounds are terpenoids such as the antifungal products plagicosins A-N, or alkaloids such as plagiochianins A-B from the Chinese liverworts *P. fruticosa* Mitt. and *P. duthiana* Steph., respectively [18,19]. However, the leading product isolated from *Plagiochila* species is without doubt the sesquiterpenoid plagiochilin A, first isolated from several *Plagiochila* species in the 1970s, together with its congeners plagiochilins B and C, and precursors plagiochilide and plagiochilal [20]. Over the past 43 years, different analogues have been isolated leading to a series of 24 derivatives, designated plagiochilins A-to-X, and related compounds (Figure 1). The present review deals the identification of these compounds and their pharmacological properties. Information about their mechanism of action is often very limited, but important observations have been made, leading to the identification of potential targets for some of these compounds, in particular for the leader product plagiochilin A (Figure 2).

Several scientific databases (mostly PubMed, Science Direct and Scopus) and internet search engines (Google, Bing) were used to execute a systematic search of the existing literature, considering all publications published up until January 2023, without any language restriction. Databases were queried using specific keywords such as “bryophytes”, “*Plagiochila*”, “natural products”, “aromadendrane”, and “plagiochilin”. The articles were searched using a Boolean logic operator (and/or/not) combined with Medical Subject Headings (MeSH) terms and keywords. The relevance of the collected articles was determined (individual expertise), and then the data were extracted and analyzed.

## 2. Discoveries of the Plagiochilins

**Figure 3:**
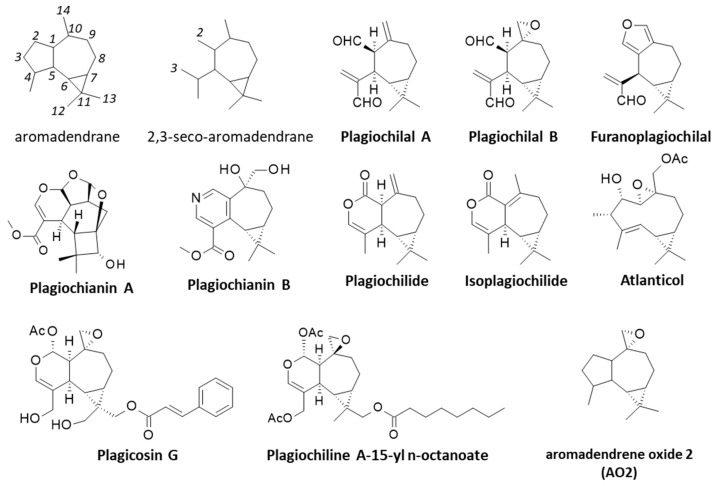
Structures of the aromadendrane and 2,3-seco-aromadendrane scaffolds (with the numbering scheme), and other products isolated from *Plagiochila* species and structurally related to the plagiochilins.

It all started in 1978 (Figure 1) when Asakawa and coworkers reported the isolation of the sesquiterpene plagiochilin A from *P. yokogurensis* Stephani, as the epoxide counterpart of plagiochilide (Figure 3) [21]. The two products with a seco-aromadendrane skeleton have been found also in *P. fruticosa* [22]. The aromadendrene scaffold is not rare in plants. Aromadendrene is an antibacterial product found in the medicinal plant *Lophostemon suaveolens* [23] and in the common hop (*Humulus lupulus* ‘Nordbrau’) [24], for example. However, epoxide derivatives with an aromadendrene scaffold are quite rare. A compound designated aromadendrene oxide (an epoxide derivative) has been isolated from the plants *Tetradenia riparia* (Hochst.) Codd, (Lamiaceae) and *Kickxia aegyptiaca* (L.) N. (Plantaginaceae) and shown to display antibacterial effects [25,26]. The 2,3-seco-aromadendrane unit is typical of *Plagiochila* species (Figure 3). The terpenoids plagiochilin A from *P. yokogurensis* and plagicosin G from *P. fruticosa* [18], and the alkaloids plagiochianins A and B from *P. duthiana* are all seco-aromadendrane derivatives [19] (Figure 3).

Following the isolation of plagiochilin A from *P. yokogurensis*, Asakawa and coworkers discovered a variety of seco-aromadendrane-type sesquiterpenoids from several *Plagiochila* species, including plagiochilins C, D, E and F in *P. asplenioides,* plagiochilins A and C in *P. semidecurrens* [27] and related products in other *Plagiochila* species, such as *P. fructicosa*, *P. ovalifolia*, and *P. porelloides* [28]. Notably, they identified plagiochilins A and B from *P. hattoriana* [21] and from *P. pulcherrima* [29], followed with plagiochilin C from both *P. ovalifolia* and *P. asplenioides*. They also identified plagiochilins D, E and F from *P. asplenioides*, together with a few related compounds such as furanoplagiochilal, plagiochilal A-B and plaigiochilide [28] (Figure 3). Similarly, plagiochilins A and B have been identified from *P. semidecurrens* [30] and *P. diversifolia* [31]. Plagiochilin A was found also in *P. elegans*, together with isoplagiochilide [32].

The species *P. porelloides* has afforded 2,3-secoaromadendrane-type sesquiterpene esters derived from plagiochilin D [33]. Seco-aromadendrane-type sesquiterpenoids are considered as chemosystematic markers in the Plagiochilaceae [34]. In the early 1980s, studies were essentially concerned with the isolation of the compounds and their structural characterization. Nevertheless, the insecticidal activity of plagiochilin A was evidenced early on, notably its capacity to inhibit the feeding of the army worm *Spodoptera exempta* (Fabricius, 1775) [35]. Plagiochilin A exhibits a strong pungent taste; it can be converted into plagiochilal B and furanoplagiochilal upon exposure to human saliva [36].

**Figure 4:**
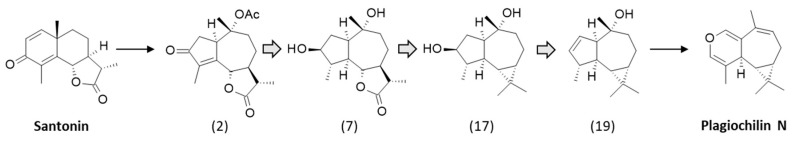
The total synthesis of plagiochilin N has been achieved from sesquiterpene lactone santonin (in fact from O-acetylisophotosantonin (2), obtained by photochemical rearrangement of santonin). The total synthesis included 16 steps. Only selected key intermediates are shown here. See [42] for the detailed synthesis which confirmed the stereochemistry of the compound.

Plagiochilin G was isolated subsequently from *P. ovalifolia*, together with plagiochilins H and I from *P. yokogurensis* [37]. Plagiochilin H is a close analogue of plagiochilin C, whereas plagiochilins G and I are structurally close to plagiochilins A-B (Figure 2). *P. ovalifolia* provides a rich source of seco-aromadendrane sesquiterpenoids, notably ester derivatives of plagiochilin A endowed with cytotoxic properties [38]. From this species, the isolation and structural characterization of plagiochilins C and N were reported, together with the derivative acetoxyisoplagiochilide [39,40]. Later, a total synthesis of plagiochilin N was proposed from the natural precursor santonin, a common anthelmintic sesquiterpene lactone readily available [41] (Figure 3). It is a long and difficult synthesis (16 steps), useful to confirm the stereochemistry of plagiochilin N [42] (Figure 4). Plagiochilin N is the only compound in the series for which a total synthesis has been proposed. All the other plagiochilins are natural products. There is a need for efficient syntheses of compounds related to plagiochilin A. Plagiochilins J and K have been isolated from *P. *fruticosa** in 1991, together with the sesquiterpene dialdehyde plagiochilal B which is considered as the precursor for these two plagiochilins [43]. Plagiochilins L and M were described four years later, but they derive from a totally distinct liverwort species, *Heteroscyphus planus*, which is however chemically similar to *Plagiochila* species. It contained also plagiochilin C. *Plagiochila* and *Heteroscyphus* belong to two families of the same sub-order (Lophocoleineae). The presence of these compounds may be more widespread between the species. The two compounds L and M bear the seco-aromadendrane skeleton typical of the product family [44]. Plagiochilin M has been isolated also from the andine species *Plagiochila tabinensis* Steph. [45].

Then, five other plagiochilins (O-P-Q-R-S) were discovered from a diethyl ether extract of the Colombian liverwort *P. cristata* (O-P-Q), from the *P. ericicola* (R) and from a dichloromethane extract prepared from an axenic culture of *P. adianthoides* (S) [46]. Plagiochilin P can also be found in *P. asplenioides* [47]. The species *P. cristata* and *P. ericicola* also contained plagiochilins C and H which are both structurally close to plagiochilin O, whereas plagiochilin R is a close analogue to plagiochilin B (Figure 2). Plagiochilins T and U were disclosed two years later, isolated from a specimen of *P. carringtonii* collected in Scotland [48]. They correspond to C-13 oxidation products derived from plagiochilin C (Figure 1). This particular *Plagiochila* species, a leafy liverwort officially named *Plagiochila carringtonii* (Balf. ex Carrington) Grolle (commonly called Carrington’s Featherwort) is well distributed in Scotland and Ireland [49]. It is distinct from the species *P. atlantica* also abundant in the West of Scotland and which was shown to contain plagiochilin C and a structurally close product designated atlanticol (Figure 3) [50] (not to be confused with another product also called atlanticol, an alkaloid from the Rutaceae *Spiranthera atlantica* [51]). Compound plagiochilin V has been rarely mentioned. It is cited in a single publication about the species *P. porelloides* from Changbai mountain in China [52]. This species has been used to isolate 2,3-secoaromadendrane-type esters derived from plagiochilin D [32]. It has been used recently as a model to study desiccation tolerance [53]. To our knowledge, the chemical structure of plagiochilin V, proposed 25 years ago, has not been confirmed. The series ends up with plagiochilins W-X, both isolated from a sample of *P. asplenioides* collected in Germany, together with the acetylated hemiacetal plagiochilin H [54].

Altogether, the plagiochilins represent a series of 24 natural products isolated from diverse *Plagiochila* species between 1978 and 2005 [55,56,57]. A few other 2,3-seco-aromadendrane derivatives were isolated subsequently but named differently, such as plagicosin G with a phenylpropanoyloxy side chain (Figure 3), isolated from *P. fruticosa* [18]. The majority of these plagiochilin compounds has been structurally characterized, but pharmacologically neglected. In rare cases, individual properties have been reported, as described below.

## 3. Pharmacological Properties and Mechanism of Action of the Plagiochilins

### 3.1. Pharmacological Properties of Plagiochilins A and C

The pharmacological effects of the plagiochilins have been rarely investigated. Nevertheless, a few types of bioactivities have been reported with plagiochilins A and C. The data in Table 1 illustrate the potential of the two compounds, but there is no systematic study comparing activities of all compounds, or activities of a given compound across multiple indications or pathologies. Initially, it was shown that plagiochilin A displays a modest antifeedant action against the armyworm *Spodoptera exempta* Walker (Lepidoptera: Noctuidae) which is an episodic migratory pest of cereal crops in sub-Saharan Africa. The level of activity is quite modest [35]. Another study has investigated the insecticidal activity of natural products isolated from *P. diversifolia*, but in that case the only plagiochilin tested was plagiochilin B and no activity was reported. In this case, a marked insecticidal action was observed with another epoxide-containing compound called fusicogigantone B, a fusicoccane-type diterpenoid [31]. Both fusicogigantones A and B, isolated from *P. bursata* and *P. diversifolia*, respectively, have been shown to inhibit the growth of another species of Lepidoptera (*Spodoptera frugiperda*) [17,31]. Plagiochilin A was shown to display a noticeable antiprotozoal activity, reducing the growth of the amastigote form of *Leishmania amazonensis*, with an IC_50_ value of 7.1 µM. However, the level of activity is quite modest compared to that of the control drug amphotericin B (IC_50_ = 0.13 µM). In the same study, no activity was observed against the fungus *Mycobacterium tuberculosis* [13].

In a more interesting way, plagiochilin A was characterized as an antiproliferative agent, reducing the growth of different cultured cancer cell lines. Both plagiochilins A and C display marked antiproliferative activities and there are known options to further increase their anticancer potency. An option is to introduce a methoxy group at position C-3, as observed with the derivative methoxyplagiochilin A2 which has been shown to be more potent than plagiochilin C against H460 lung cancer cells (IC_50_ = 6.7 and 13.1 µM, respectively) [58]. Another option is to introduce a side chain at the C-14/C-15 position, either an octanoyl side chain or a dodecadienoate side chain, for example. In both cases, the resulting compounds were found to be 60 times more potent against P-388 leukemia cells than the parent compound plagiochilin A [38]. The extraordinary potency of plagiochilin A-15-yl n-octanoate (Figure 3) raises questions (solubility, stability) and opens perspectives. The octanoate moiety may serve only as a “lipophilic carrier” (bioavailability enhancement), and may not be directly implicated in the target interaction. Novel C-12/C-13-substituted derivatives of plagiochilin A should be designed.

**Figure 5:**
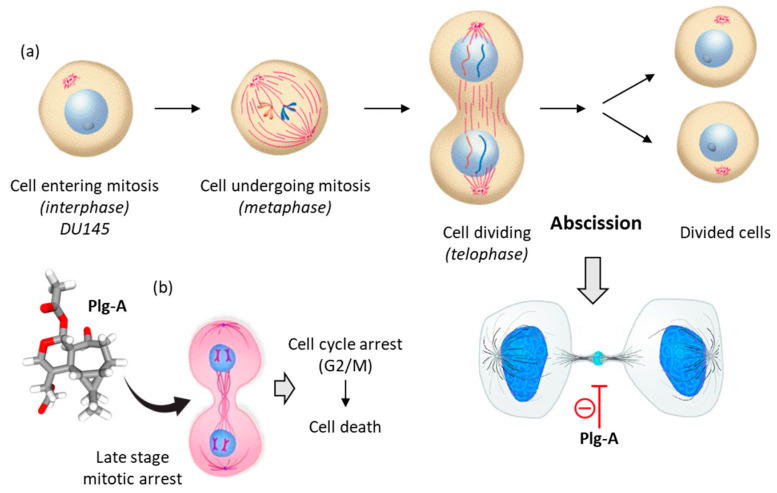
Mechanism of action of plagiochilin A (Plg-A). (**a**) The cell division process, to illustrate DU145 prostate cancer cells undergoing mitosis. Prior to cell division, at the telophase stage of mitosis, the two cells are connected by an intercellular bridge (with a central midbody). Plg-A inhibits cell division by preventing completion of cytokinesis, particularly at the final abscission stage. (**b**) Inhibition of the late stage of cytokinesis leads to cell cycle arrest (G2/M) and subsequently to inhibition of cell colony formation and induction of cell death [59].

Plagiochilin A exhibits antiproliferative activities against different types of cancer cells. The growth inhibition GI_50_ values ranged from 1.4 to 6.8 µM with a range of tumor cell lines, including prostate (DU145), breast (MCF-7), lung (HT-29) and leukemia (K562) cells for example [13]. The level of activity against DU145 prostate cancer cell is interesting (GI_50_ = 1.4 µM) because it is superior to that observed with the reference anticancer drug fludarabine phosphate (GI_50_ = 3.0 µM). The sensitivity of prostate cancer cells toward plagiochilin A warranted further investigation. In 2018, Bates and coworkers analyzed the effect of plagiochilin A on the cell cycle progression of DU145 cells and their capacity to complete cytokinesis, the part of the cell division process during which the cytoplasm of a single eukaryotic cell divides into two daughter cells. Interestingly, it was observed that the compound (at 5 µM for 24–48 h) could block cell division by preventing completion of cytokinesis, and thereby inducing cell death [59]. The treated DU145 cells accumulated at the G2/M phase, notably cells still connected with intercellular bridges, corresponding to a late cytokinesis stage, the so-called membrane abscission stage (stained with an α-anti-tubulin antibody). The compound induced specific mitotic figures and reduced significantly the number and size of DU145 cell colonies. The failure of the cells to complete cytokinesis triggered apoptosis [59]. Altogether, these data indicated that plagiochilin A exerts an effect on the cytoskeleton, with a rearrangement of α-tubulin characteristic of cytokinetic membrane abscission, which is a spatially and temporally regulated process [59] (Figure 5).

### 3.2. Hypothesized Mechanism of Action of Plagiochilin A

**Figure 6:**
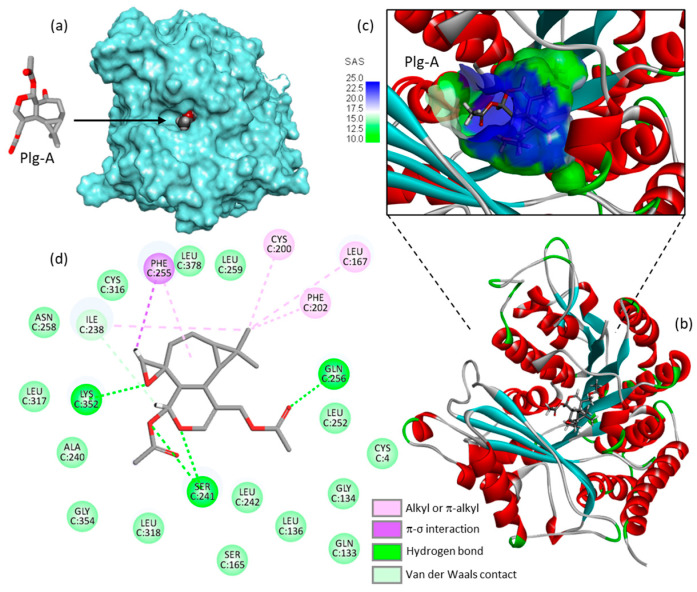
Molecular model of plagiochilin A (Plg-A) bound to the pironetin site of α-tubulin (PDB: 5FNV). (**a**) Plg-A fits into a central, deep cavity of the protein. (**b**) Ribbon model of α-tubulin with bound Plg-A, with α-helices (in red) and β-sheets (in cyan). (**c**) A close-up view of Plg-A inserted into the binding cavity, with the solvent-accessible surface (SAS) surrounding the drug binding zone (color code indicated). (**d**) Binding map contacts for Plg-A bound to α-tubulin (color code indicated). The docking model was kindly provided by Prof. Gérard Vergoten (University of Lille, France). The docking analysis was performed as recently described [65].

The mechanics implicated in the regulation of abscission is relatively well-known. This process leads to the physical cut of the intercellular bridge which connects two daughter cells and concludes cell division. The process is tightly regulated in cells, with intervention of multiple protein effectors including different kinases (e.g., PLK4, Aurora B) and proteins containing microtubule-interacting and trafficking (MIT) domains [60,61,62]. The process opens perspectives to comprehend the mechanism of action of plagiochilin A. the compound may target MIT-containing proteins, or more directly it may associate with alpha-tubulin during mitosis, for example. There exist small molecules which induced cytokinesis failure at the point of abscission, such as a series of dynamin GTPase inhibitors called dynoles [63,64]. These products induce apoptosis following cytokinesis failure, as observed with plagiochilin A. Therefore, we can imagine that the natural product acts as an inhibitor of termination of cytokinesis (abscission) by blocking one or several proteins implicated in the process, or by directly altering the microtubule-organizing center which recruits α- and β-tubulins for microtubule nucleation. In this context, one of the potential mechanisms could be a direct binding of the compound to α-tubulin, in particular to the pironetin-binding site which is known to accommodate compounds bearing a dihydro-pyrone moiety [65,66]. This moiety can be found in plagiochilin Q, for example, and recently, we have shown that natural products with a 5,6-dihydro-α-pyrone unit (cryptoconcatones) can function as α-tubulin-binding agents [67]. Based on these considerations, we have initiated binding studies of plagiochilins to α-tubulin and the first information obtained by molecular docking look interesting. For the docking analysis, the high-resolution crystal structure of the reference product pironetin (a dihydropyrone derivative with an α,β-unsaturated lactone acting as a plant growth regulator) bound to α/β-tubulin dimer was used as a template (PDB: 5FNV) and the binding of plagiochilin A to the pironetin site was modeled. Apparently, plagiochilin A could form stable complexes with α-tubulin, via binding to the pironetin site, as represented in Figure 6. These are preliminary, but promising information. We are now comparing various plagiochilins for their capacity to bind to α-tubulin, using molecular modeling. The mechanism whereby plagiochilin A specifically blocks abscission warrant further investigation. The compound is an atypical inhibitor of cytokinesis. The panoply of plagiochilins shall be further exploited to identify the best inhibitors and to delineate the structure–activity relationships in the series. The modeling analysis shall help also to delineate the mechanism of action of other aromadendrane derivatives, such as the related natural products hanegokedial, ovalifolienal, ovalifolienalone (from *P. semidecurrens*) and others [30,68].

## 4. Discussion

Bryophytes are known to produce bioactive compounds with a broad range of therapeutic potential [69]. For example, extracts prepared from a range of mosses and liverworts have been screened recently for their anti-inflammatory properties. Two particular species, *Dicranum majus* Sm. And *Thuidium delicatulum* (Hedw.) Schimp., were found to inhibit production of nitric oxide in lipopolysaccharide-stimulated Raw 264.7 murine macrophages [70]. In this study, an extract of *Plagiochila asplenioides* (L.) Dumort. Was tested but it did not show a marked activity. Other bryophyte extracts have revealed antioxidant and/or antimicrobial activities [71,72]. The use of extracts from *Plagiochila* species is relatively rare. However, there are noticeable exceptions, for example, the use of methanol extract of the liverwort *Plagiochila beddomei* Steph. which has revealed marked antimicrobial activities against a wide group of bacteria and fungi, including the pathogen *Candida albicans* (MIC = 0.75 mg/mL). The extract contained flavonoids, saponins, tannins and phenols [11,12,73]. In most cases, bioactivities with bryophytic extracts have been attributed to the presence of flavonoids, terpenoids or alkaloids, such as macrocyclic bibenzyls and bis(bibenzyls), and many sesqui- and diterpenoids [74,75,76,77,78,79]. However, *Plagiochila* species and plagiochilins are rarely evoked.

The different *Plagiochila* species mentioned here produce 2,3-secoaromadendrane-type sesquiterpenes, chiefly the cytotoxic product plagiochilin A [56,80]. This compound is considered as being responsible for the pungent and bitter taste of *Plagiochila* liverworts, at least in part [80]. However, it is also a robust anticancer agent, insufficiently considered despite its very innovative mechanism of action as a late-stage cytokinesis inhibitor. Whether the compound target α-tubulin or other proteins implicated in membrane abscission remains to be determined experimentally. However, whatever the exact target, the mode of action of the compound is particularly attractive and inspiring. There are not many products acting at the abscission checkpoint, and it is probably the only natural product known to regulate cytokinetic abscission. Plagiochilin A could serve as a useful tool to dissect the abscission mechanism which is a multi-step process which influences cell fate and tissue growth [81,82]. There is a need for pharmacological tools to dissect the mechanism of abscission of nascent daughter cells, to stop or halt the process and to study the implication of specific protein complexes and cellular structures, such as midbody proteins and the midbody organelle [83]. There is a rearrangement of α-tubulin and actin, and important cytoskeleton modification during abscission. The process can be modulated with the use of histone deacetylase (HDAC) inhibitors [84], actin polymerization inhibitors [85], and various kinase inhibitors [86]. New tools are needed to dissect the process, notably to help understanding the function of the residual midbody remnant generated at the end of the process and which affects cell fate and tumorigenesis [87]. Plagiochilin A has the capacity to induce an accumulation of cancer cells (at least DU145 prostate cancer cells) connected by intercellular bridges, probably via a selective action on the sequential assembly of the endosomal sorting complexes required for transport (ESCRT) machinery [59]. The compound affords a useful tool to dissect the process, to study ESCRT assembly and regulation. In parallel, the use of high-content phenotypic screens focused on cytokinesis would be useful to further identify the targets of the compound and the fine tuning of its mechanism of action [88].

Plagiochilin A deserves further studies as an anticancer agent owing to its mechanism of action and its level of activity. It could be a useful starting point to elaborate more potent analogues because there are strategies to enhance the antiproliferative potency of plagiochilin A, notably via C-12/C-13 substitution [38]. There are also many naturally occurring derivatives (plagiochilins, plagiochilide, plagiochilal, and others) which could be exploited to determine structure–activity relationships. Moreover, the chemical diversity of the natural products can be enhanced upon biotransformation with microorganisms. For example, plagiochilide can be chemically modified to afford 12-hydroxyplagiochilide and plagiochilide-12-oic acid in the presence of the fungus *Aspergillus niger* [22]. The difficulty is to obtain the compounds. There is a possibility to produce biomass under laboratory conditions through bryo-reactors and molecular farming (thus decreasing pressure for natural populations) [89], but the subsequent purification of natural products would not be an easy ride. Axenic cultures of *Plagiochilla* have been developed in rare cases only, notably for *P. arctica* Bryhn and Kaal. [90]. 

Apart from plagiochilin N for which a total synthesis has been reported [40], the other compounds have to be isolated from plants or novel syntheses have to be designed for these compounds. An alternative option is to access the related compound called aromadendrene oxide 2 (AO2) which is an analogue of plagiochilin A (Figure 3). This sesquiterpene can be found in essential oils from different plants [25,91,92]. The compound has been shown to induce apoptosis of skin epidermoid A431 cancer cells, via activation of the mitochondrial pathway [93,94]. Its activity further supports the interest for plagiochilin A and more globally the use of the 2,3-secoaromadendrane as a template to design novel anticancer agents. There are other 2,3-secoaromadendrane-type sesquiterpenoids of interest, with an unknown mechanism of action, such as the two products psilosamuiensins A and B isolated from the broth of the psychoactive fungus *Psilocybe samuiensis* [95]. This family of terpenoids with a 2,3-secoaromadendrane scaffold should be investigated further as a source of anticancer agents. More generally, this work also underlines the interest of bryophytes as a source of bioactive compounds, and anticancer agents in particular. In recent years, different bryophytes species have revealed marked antitumor effects, including a capacity to kill chemo-resistant cancer stem cells [71,96]. The use and study of bryophytes, in particular liverworts (Hepaticae or hepatics), shall be encouraged [97,98]. Liverworts lack roots, seeds, fruit and flowers, but they do not lack major interest as a source of bioactive compounds. It is time to get these simple, single cellular plants back into the limelight, and in particular the *Plagiochila* group of leafy liverworts which can offer an array of captivating compounds. 

## 5. Conclusions

The analysis shed light on a little-known family of 24 terpenoids designated plagiochilins A-to-W, isolated from various Plagiochila species. Leafy liverworts of the genus *Plagiochila* produce a variety of seco-aromadendrane-type sesquiterpenes, among which plagiochilin A is a lead product endowed with marked antiproliferative activities against cancer cells. This epoxide-containing natural product functions has been characterized as an inhibitor of the termination phase of cytokinetic abscission, a somewhat unique action at the origin of its capacity to delay cell cycle progression and to induce cell death. This atypical mechanism of action makes plagiochilin A an interesting tool to study the abscission process and a lead compound to design more potent analogues. There are known options to increase the potency of the compound. Preliminary structure–activity relationships have been delineated in the plagiochilin series. The *Plagiochila* genus of briophytes deserves further studies as a source of bioactive compounds.

## Figures and Tables

**Table 1:** Bioactivities reported with plagiochilins.

Compounds	Bioactivities	Tests/Species	End Points	Ref.
Plagiochilin A	Antifeedant	African armyworm *Spodoptera exempta*	Activity observed at 1–10 ng/cm^2^	[35]
Plagiochilin A	Antiparasitic	*Leishmania amazonensis* axenic amastigotes	IC_50_ = 7.1 µM	[13]
Plagiochilin A	Antiparasitic	*Trypanosoma cruzi* trypomastigotes	MIC = 14.5 µM	[13]
Plagiochilin A	Anti- proliferative	P-388 murine leukemia cells	IC_50_ = 3.0 µg/mL	[38]
Plagiochilin A	Anti- proliferative	A172 glioblastoma cells	IC_50_ = 19.4 µM.	[56]
Plagiochilin-A-15-yl n-octanoate	Anti- proliferative	P-388 murine leukemia cells	IC_50_ = 0.05 µg/mL	[38]
Plagiochilin C	Antiplatelet	Inhibition of arachidonate-induced rabbit platelet aggregation	95% and 45% inhibition at 100 and 50 µg/mL, respectively.	[32]
Plagiochilin C	Anti- proliferative	A172 glioblastoma cells	IC_50_ = 4.3 µM	[58]

## Data Availability

Data are contained within the article.
